# Comparative Cardiovascular Outcomes of Fimasartan and Calcium Channel Blockers in Hypertension: A Nationwide 3-Year Follow-Up Cohort Study

**DOI:** 10.3390/jcm15197669

**Published:** 2026-10-03

**Authors:** Yuran Ahn, Ji In Lee, Han Saem Jeong, Hong-Seok Lim, Jin-Oh Choi, Sang-Ho Jo, Soon Jun Hong, Hyo-Suk Ahn, Hae Ok Jung, Wang Soo Lee, Hun-Jun Park

**Affiliations:** 1 Division of Cardiology, Department of Internal Medicine, Uijeonbu St. Mary’s Hospital, The Catholic University of Korea, Seoul 06591, Republic of Korea; niceayr@naver.com (Y.A.); alaco0502@gmail.com (H.-S.A.); 2 Department of Clinical Research Design and Evaluation, Samsung Advanced Institute for Health Sciences & Technology (SAIHST), Sungkyunkwan University, Seoul 03063, Republic of Korea; lj1199@naver.com; 3 Dr. Jeong’s Heart Clinic, Jeonju 54985, Republic of Korea; mdhansaem@gmail.com; 4 Department of Cardiology, Ajou University School of Medicine, Suwon 16499, Republic of Korea; camdhslim@ajou.ac.kr; 5 Division of Cardiology, Department of Medicine, Heart Vascular Stroke Institute, Samsung Medical Center, Sungkyunkwan University School of Medicine, Seoul 06351, Republic of Korea; jinoh.choi@samsung.com; 6 Division of Cardiology, Department of Internal Medicine, Hallym University Sacred Heart Hospital, Anyang 14068, Republic of Korea; soph5@hallym.or.kr; 7 Department of Cardiology, Cardiovascular Center, Korea University Anam Hospital, Seoul 02841, Republic of Korea; psyche94@gmail.com; 8 Division of Cardiology, Department of Internal Medicine, Seoul St. Mary’s Hospital, The Catholic University of Korea, Seoul 06591, Republic of Korea; 9 Division of Cardiology, Department of Internal Medicine, College of Medicine, Chung-Ang University Hospital, Seoul 06973, Republic of Korea

**Keywords:** hypertension, fimasartan, calcium channel blocker, angiotensin receptor blocker, major adverse cardiovascular events

## Abstract

**Background:** Fimasartan is an angiotensin II receptor blocker (ARB) that was developed in Korea. Although ARBs and calcium channel blockers (CCBs) are both recommended first-line antihypertensive drug classes, real-world outcome data comparing fimasartan specifically with CCBs remain limited. **Methods:** We analyzed Korean National Health Insurance Service data for adults aged ≥20 years with hypertension and no prior cardiovascular disease. The primary inferential analysis was treatment-naïve new users of fimasartan or CCB monotherapy using a 30-day landmark design, with outcome follow-up beginning on day 31. The primary endpoint was 3-year major adverse cardiovascular events (MACEs), defined as all-cause death, cardiac death, nonfatal myocardial infarction, or nonfatal stroke. Multivariable Cox models were used for the primary analysis, while broader overall-cohort and propensity-score-matched analyses were considered secondary. **Results:** In the primary treatment-naïve new-user analysis, fimasartan was associated with a numerically lower MACE risk than CCBs, although the 95% confidence interval included the null value (adjusted HR 0.895, 95% CI 0.796–1.006; *p* = 0.0628). In secondary analyses of the broader overall cohort, fimasartan was associated with a lower adjusted MACE risk (HR 0.851, 95% CI 0.815–0.890), and the association remained evident after propensity-score matching (HR 0.675, 95% CI 0.653–0.699). Exploratory comparisons showed nearly identical crude MACE rates for fimasartan+CCB and fimasartan+thiazide regimens (3.46% vs. 3.47%). **Conclusions:** In this nationwide observational cohort, the primary treatment-naïve new-user analysis showed a numerically lower 3-year MACE risk with fimasartan than with CCBs, although the confidence interval included the null value. Stronger associations observed in secondary analyses of the broader cohort should be interpreted cautiously because residual confounding and selection bias cannot be excluded.

## 1. Introduction

Hypertension remains one of the most important modifiable contributors to cardiovascular morbidity and mortality worldwide [1,2]. Blood pressure lowering reduces major cardiovascular events across diverse populations [3,4], and contemporary guidelines recommend several first-line drug classes, including renin-angiotensin system inhibitors, CCBs, and thiazide or thiazide-like diuretics [5,6,7]. In clinical practice, however, the choice between an ARB and a CCB often depends on patient characteristics, comorbidities, tolerability, and local prescribing patterns rather than direct comparative outcome evidence.

Prior Korean nationwide data suggested that ARB treatment as a class was associated with fewer cardiovascular events than CCB treatment among hypertensive patients without established cardiovascular disease [8]. Randomized trials and meta-analyses comparing ARBs or other renin-angiotensin system blockers with CCB-based regimens have yielded mixed results, with outcomes influenced by population risk, blood pressure differences, and the specific drugs being compared [9,10,11,12]. These data do not establish whether the findings observed for the ARB class can be applied to every ARB or to fimasartan specifically. Fimasartan is a pyrimidinone-derived ARB with high affinity for the angiotensin II type 1 receptor and favorable blood pressure-lowering efficacy in Korean patients with hypertension [13,14,15,16,17,18,19,20,21]. A recent nationwide cohort study reported that fimasartan, when combined with a CCB, was associated with a lower transition rate to triple antihypertensive therapy than other ARBs combined with CCBs [22]. However, cardiovascular event data comparing fimasartan monotherapy with CCB monotherapy in a large real-world hypertensive population remain sparse. We therefore evaluated 3-year cardiovascular outcomes associated with fimasartan and CCBs using the Korean National Health Insurance Service (NHIS) database, with particular attention to measured confounding, treatment-naive and treatment-maintenance patterns, and the limits of causal interpretation.

## 2. Materials and Methods

### 2.1. Data Source and Study Design

This retrospective nationwide cohort study used data from the Korean NHIS, a mandatory public insurance system covering nearly the entire Korean population. The NHIS database includes demographic information, insurance eligibility, diagnosis codes, prescription claims, procedures, death information, and national health screening data [23]. The study period covered index hypertension identification in 2018 and follow-up through 31 December 2020. The manuscript was prepared in accordance with reporting principles for observational cohort studies, emphasizing transparent exposure definitions, outcome ascertainment, measured confounding control, and limitations of causal interpretation.

### 2.2. Study Population

Adults aged ≥20 years with hypertension, defined by International Classification of Diseases, Tenth Revision (ICD-10) code I10 in 2018, were screened. The baseline look-back period was 2012–2017. We excluded patients with prior heart failure (I50, I110, I130, I132), unstable angina (I20.0), angina pectoris (I20.1, I20.8, I20.9), atrial fibrillation (I48), stroke (I60-I66), peripheral artery disease (I70-I79, I70.2-I70.3, I70.9, I73.1, I73.8-I73.9, and related NHIS procedure codes M6597, M6605, M6613, M6632, M6620, O0161-O0171, O1643-O1644, and O1645-O1646), or prior myocardial infarction (MI). Prior MI was assessed as a separate exclusion criterion so that the final cohort was free from MI as well as the other prespecified cardiovascular diseases. Patients without prescriptions for angiotensin-converting enzyme inhibitors, ARBs, CCBs, or thiazides during follow-up were excluded. Patients receiving combination antihypertensive therapy at baseline were excluded from the main monotherapy comparison. The diagnosis and procedure codes used for exclusion criteria and outcome ascertainment are summarized in Supplementary Table S6.

**Figure 1:**
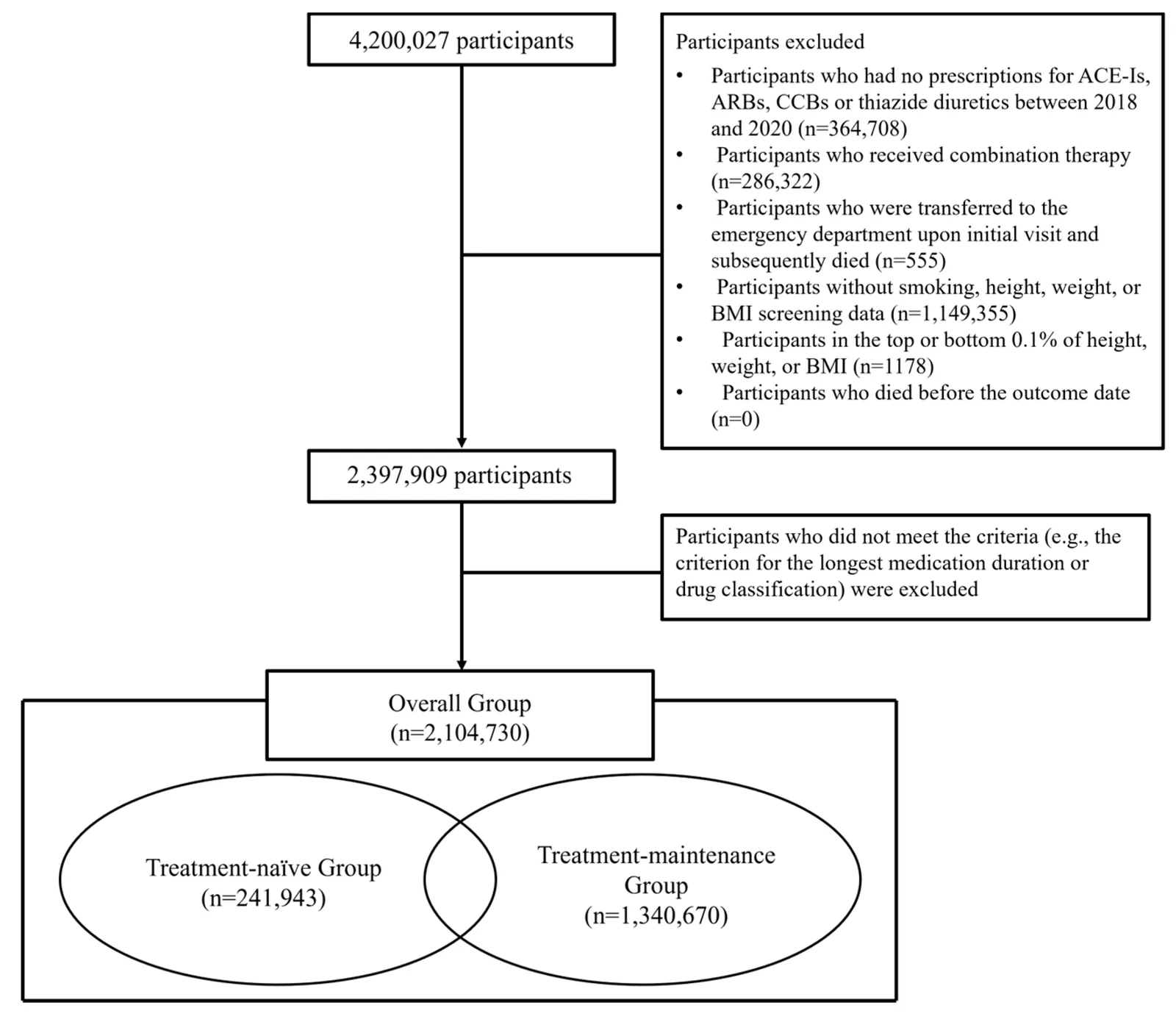
Flow diagram of the study population selection.

Patients transferred to an emergency department and dying during the first visit were excluded to avoid assigning outcomes that occurred before meaningful drug exposure. We also excluded patients with missing smoking, height, weight, or body mass index (BMI) information and those with extreme height, weight, or BMI values in the upper or lower 0.1% of the distribution. Atrial fibrillation was excluded even though it was not an endpoint because it strongly alters stroke risk and often entails rate-control or anticoagulant therapy, making MACE comparisons difficult to interpret in a simple-hypertension cohort. Beta-blockers were not included as a primary comparator because their use is often driven by compelling indications such as ischemic heart disease, heart failure, or arrhythmia, many of which were exclusion criteria in this simple-hypertension cohort; including beta-blockers would therefore be expected to increase confounding by indication (Figure 1).

The primary inferential analysis was treatment-naïve new users of fimasartan or CCB monotherapy. The index date was defined as the date of the first antihypertensive prescription in 2018. To reduce exposure misclassification from transient prescriptions, patients were required to continue the index treatment for at least 30 days. A 30-day landmark design was applied, and outcome follow-up began on day 31. Analyses of the broader overall cohort were considered secondary. Treatment-maintenance analyses were considered descriptive because treatment persistence was defined using prescription information accrued during follow-up and could therefore be affected by survivor and conditioning-on-the-future selection. Additional descriptive analyses evaluated fimasartan-based combination regimens, including fimasartan+CCB and fimasartan+thiazide.

### 2.3. Treatment Definitions

Treatment-naive patients were defined as those without prior hypertension treatment before the index period whose first antihypertensive prescription in 2018 was continued for at least 30 days. The 30-day criterion was used to reduce exposure misclassification from transient prescriptions. Treatment-maintenance patients were those with a history of hypertension treatment whose primary therapy was the drug prescribed for the longest duration and covering at least 30% of the 3-year observation period, corresponding to ≥329 days. The overall cohort included treatment-naive and treatment-maintenance patients as well as patients who met the broader exposure criteria for the main comparison. These exposure definitions were intended to compare real-world treatment patterns rather than to estimate a per-protocol causal effect of uninterrupted drug use. Because the treatment-maintenance definition incorporates prescription persistence during follow-up, subgroup findings for treatment-maintenance patients were interpreted as descriptive exposure-pattern associations. The CCB group included both dihydropyridine and non-dihydropyridine agents; the distribution of individual CCB agents is provided in Supplementary Table S7.

### 2.4. Outcomes

The primary endpoint was MACE, defined as a composite of all-cause death, cardiac death, nonfatal MI, or nonfatal stroke during 3-year follow-up. Secondary endpoints included all-cause death, MI, revascularization, hospitalization for heart failure, and ischemic stroke. All-cause death was identified from death records. Cardiac death was operationalized using cardiovascular diagnosis codes associated with death or terminal cardiovascular claims, including acute MI, stroke, heart failure, sudden cardiac arrest, and other cardiovascular causes (I00-I99). Because the NHIS is a claims database, cause-specific death was not independently adjudicated. Nonfatal MI and stroke were identified using hospitalization claims and ICD-10 codes (MI: I21-I23; stroke: I60-I66; ischemic stroke: I63-I64). Hospitalization for heart failure was defined by a hospitalization claim with heart-failure diagnosis codes and concomitant diuretic treatment (Anatomical Therapeutic Chemical class C03). Dyspnea itself was not used as a separate criterion because symptom-level information is not reliably captured in claims data. Revascularization was defined by procedure records for percutaneous coronary intervention (M6551, M6552, M6561-M6567) or coronary artery bypass graft surgery (O1640, O1641, O1647-O1649, OA640, OA641, and OA647-OA649). Whenever possible, hospitalization, procedure, and medication-treatment criteria were combined to improve outcome specificity in the claims setting. Systolic and diastolic blood pressure values are reported in mmHg.

### 2.5. Statistical Analysis

Baseline characteristics are summarized as mean ± standard deviation for continuous variables and number (percentage) for categorical variables. Categorical variables were compared using the chi-square test or Fisher exact test, as appropriate. Cox proportional hazards models were used to estimate associations between treatment and time-to-event outcomes. For analyses of nonfatal outcomes, patients who died before experiencing the outcome of interest were censored at the date of death; therefore, the corresponding Cox models estimated cause-specific hazards with death treated as a competing event. Because very large sample sizes can make small absolute baseline differences statistically significant, *p* values for baseline characteristics were interpreted together with the magnitude and direction of covariate differences. To reduce measured confounding, propensity scores were estimated using multivariable logistic regression. The model included age, sex, income, diabetes mellitus, chronic kidney disease, and Charlson comorbidity index. Fimasartan users were matched to CCB users in a 1:5 ratio using a caliper of 0.008 [24]. Multivariable Cox models additionally evaluated medication category and baseline covariates, including age, sex, income, diabetes mellitus, chronic kidney disease, Charlson comorbidity index, dyslipidemia, smoking status, BMI, and the number of concomitant antihypertensive drug classes where available. E-value sensitivity analysis was used to evaluate the potential influence of unmeasured confounding based on the adjusted hazard ratio and the confidence limit closest to the null. For estimates below 1.0, reciprocal values were used for E-value calculation [25]. All tests were two-sided, and *p* < 0.05 was considered statistically significant. Analyses were performed with SAS software (version 9.3; SAS Institute, Cary, NC, USA).

### 2.6. Ethics Approval

The study was approved by the Institutional Review Board of The Catholic University of Korea, Seoul St. Mary’s Hospital (IRB No. 2021-2214-0001). The requirement for informed consent was waived because the NHIS database was de-identified according to strict confidentiality guidelines.

## 3. Results

### 3.1. Study Population and Baseline Characteristics

The primary fimasartan-versus-CCB comparison included 1,026,389 patients, of whom 934,534 received CCBs and 91,855 received fimasartan. Before matching, fimasartan users were younger than CCB users (mean age: 60.82 ± 10.56 vs. 66.23 ± 11.55 years) and were more often male (56.88% vs. 52.44%). Diabetes mellitus and dyslipidemia were more common in the fimasartan group, whereas chronic kidney disease was less common (Table 1). Baseline systolic and diastolic blood pressures were 133.17 ± 15.63 and 82.50 ± 11.01 mmHg in the CCB group and 130.73 ± 15.54 and 81.11 ± 10.98 mmHg in the fimasartan group. At 3-year follow-up, mean systolic/diastolic blood pressure was 130.83 ± 14.12/80.35 ± 9.82 mmHg in the CCB group and 128.45 ± 14.02/79.00 ± 9.84 mmHg in the fimasartan group. The absolute reductions from baseline were similar between groups (SBP, −2.34 vs. −2.28 mmHg; DBP, −2.15 vs. −2.11 mmHg for CCB and fimasartan, respectively) (Supplementary Table S3).

Propensity-score matching generated 86,694 fimasartan users and 433,479 CCB users. After matching, measured baseline characteristics were closely balanced, including age, sex, income, diabetes, chronic kidney disease, Charlson comorbidity index, dyslipidemia, smoking, height, weight, and BMI (Supplementary Table S2). Because of the large cohort size, small numerical differences could yield low *p* values; therefore, the matched baseline profile was interpreted by the overall magnitude of covariate balance rather than by *p* values alone.

### 3.2. Clinical Outcomes During 3-Year Follow-Up

**Figure 2:**
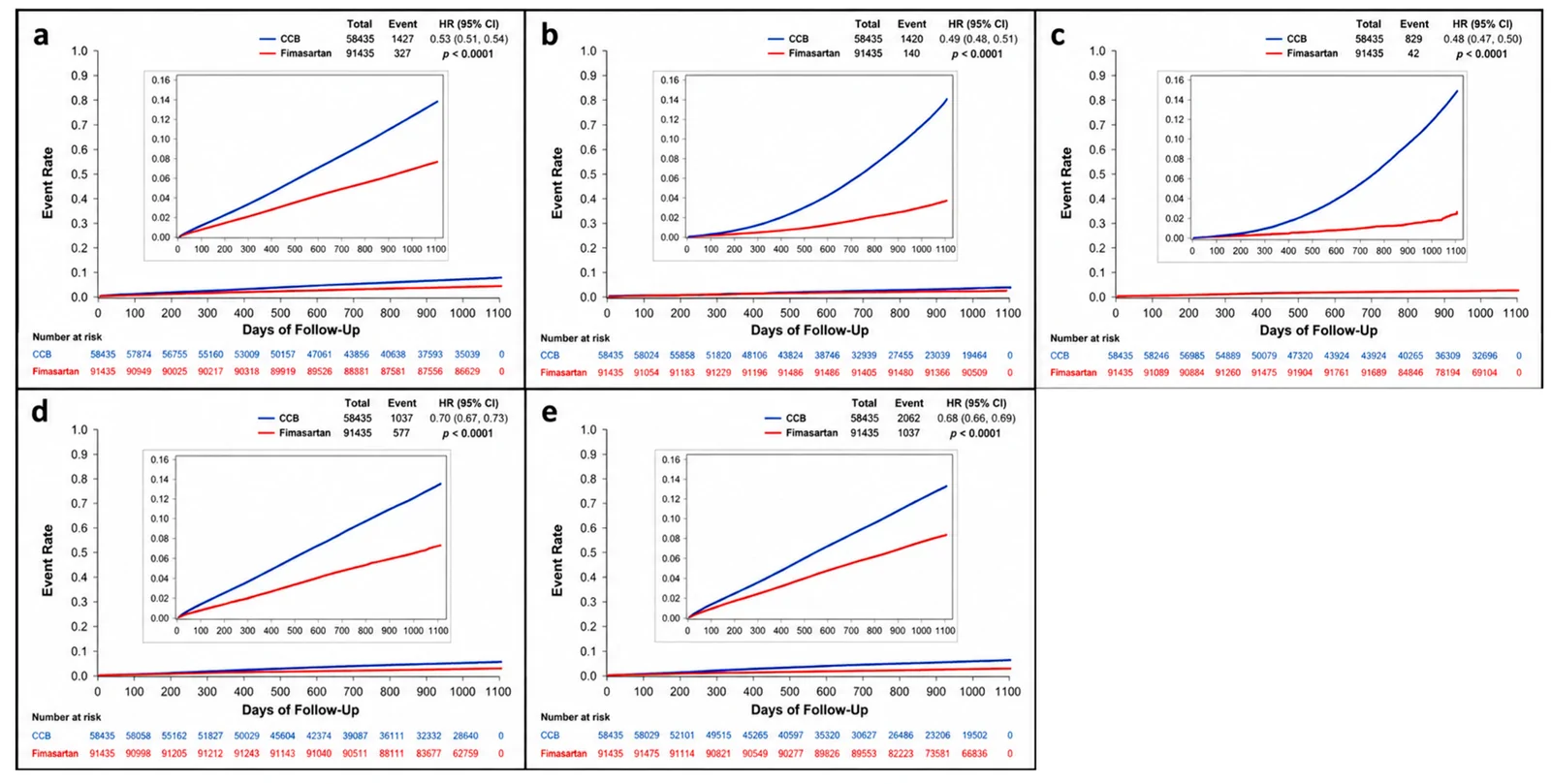
Cumulative incidence of MACE and its components during 3-year follow-up. (**a**) Major adverse cardiovascular events. (**b**) All-cause death. (**c**) Cardiac death. (**d**) Nonfatal MI. (**e**) Stroke.

During 3-year follow-up, the primary endpoint occurred in 74,217 CCB users (7.94%) and 3877 fimasartan users (4.22%) (Table 2). Each MACE component was less frequent in the fimasartan group, including all-cause death (0.37% vs. 1.51%), cardiac death (0.05% vs. 0.33%), nonfatal MI (0.56% vs. 1.11%), and nonfatal stroke (3.34% vs. 5.45%). The secondary composite outcome was also less frequent in the fimasartan group (3.15% vs. 6.74%). Time-to-event analyses showed lower cumulative MACE incidence in fimasartan users than in CCB users (hazard ratio [HR] 0.530, 95% confidence interval [CI] 0.513–0.547, *p* < 0.0001) (Figure 2).

**Figure 3:**
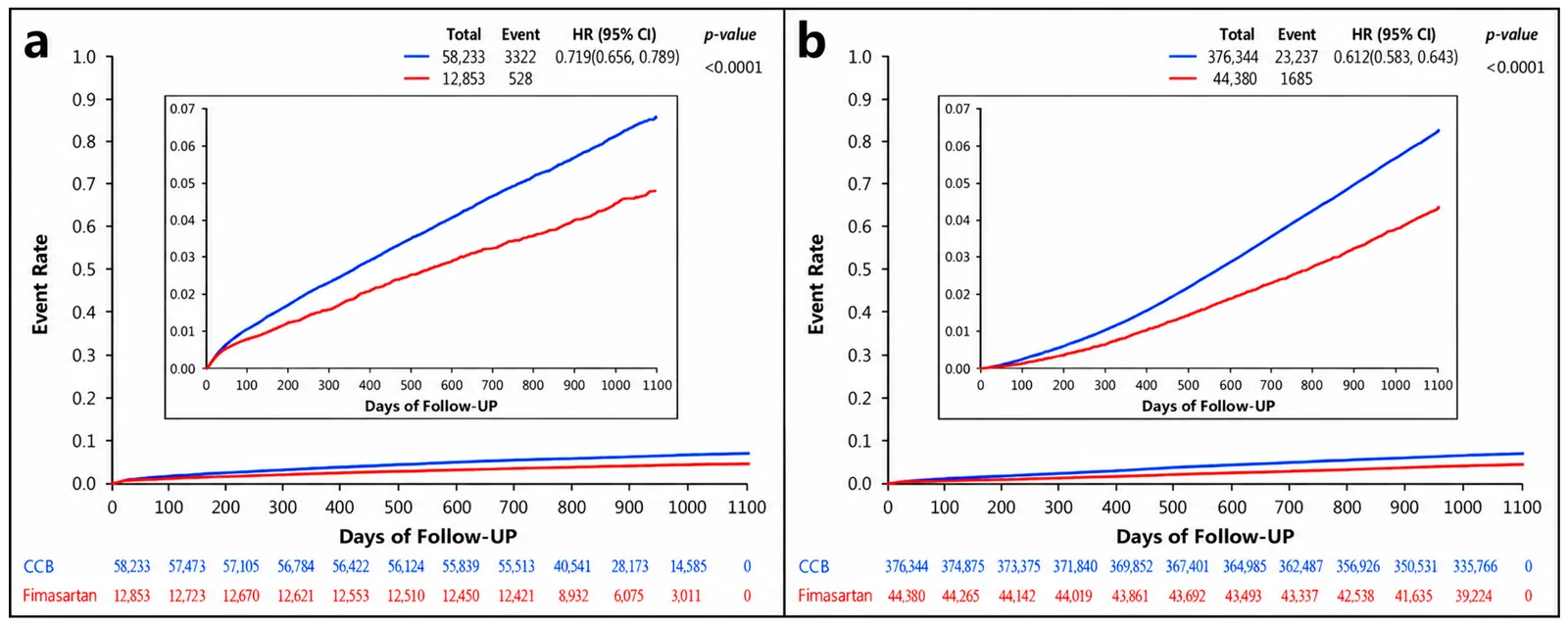
Hazard ratios for MACE by treatment-naive and treatment-maintenance status. (**a**) The treatment-naïve group. (**b**) The treatment-maintenance group.

In the primary treatment-naïve new-user analysis using a 30-day landmark design, fimasartan was associated with a numerically lower risk of MACE than CCBs, although the 95% confidence interval included the null value (adjusted HR 0.895, 95% CI 0.796–1.006; *p* = 0.0628). After propensity-score matching, event rates were also lower in the fimasartan group than in the CCB group (4.35% vs. 6.43%; Table 3), with a matched time-to-event HR estimate of 0.675 (95% CI 0.653–0.699; *p* < 0.0001). In secondary analyses of the broader overall cohort, fimasartan use remained associated with a lower MACE risk after multivariable adjustment (HR 0.851, 95% CI 0.815–0.890; *p* < 0.0001; Table 4). The treatment-maintenance analysis showed an HR of 0.840 (95% CI 0.800–0.881; *p* < 0.0001); however, this analysis was considered descriptive because treatment-maintenance status was defined using prescription persistence accrued during follow-up and may therefore be subject to survivor and conditioning-on-the-future selection (Figure 3).

**Figure 4:**
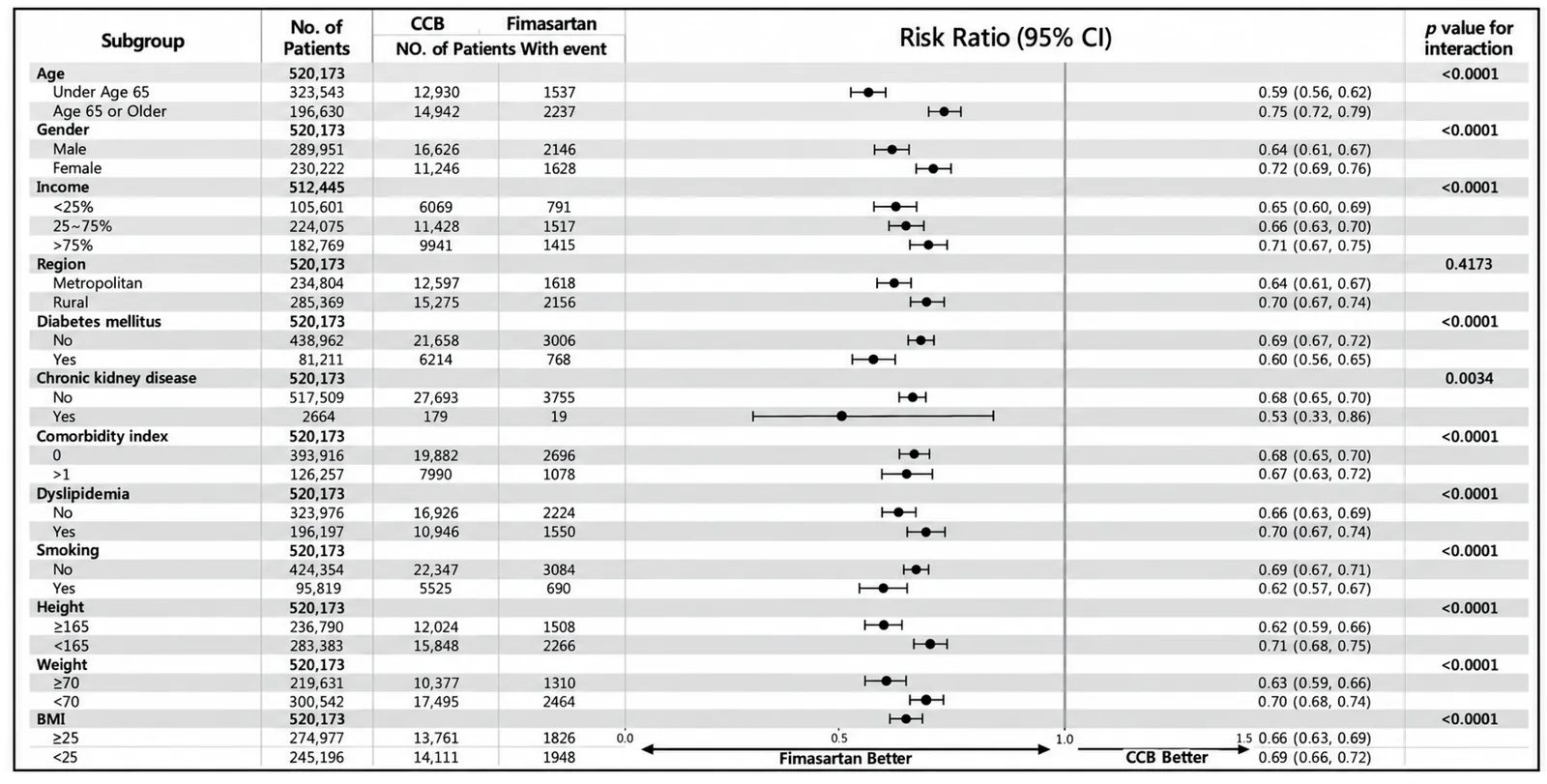
Prespecified subgroup analyses of MACE. Unadjusted risk ratios (RRs) and 95% confidence intervals were calculated from the 3-year cumulative event proportions for fimasartan versus CCB treatment.

Prespecified subgroup analyses showed unadjusted risk ratios below 1.0 across the evaluated subgroups, favoring fimasartan over CCBs. However, the magnitude of the association varied across several patient characteristics, with significant interaction *p* values for most subgroup variables, whereas no significant interaction was observed by region (*p* for interaction = 0.4173) (Figure 4).

The magnitude of the crude outcome difference was larger than that of the adjusted association, suggesting that baseline risk imbalance accounted for part of the unadjusted separation. Accordingly, crude event rates were interpreted descriptively. The treatment-naïve new-user model was considered the primary inferential analysis, whereas the broader overall-cohort and treatment-maintenance findings were interpreted as secondary and descriptive analyses, respectively. For the adjusted overall comparison, the E-value was 1.63 for the point estimate and 1.50 for the lower confidence-limit closest to the null. Thus, an unmeasured confounder associated with both treatment selection and MACE by a risk ratio of approximately 1.5–1.6-fold, beyond measured covariates, could potentially explain the observed association. This sensitivity analysis supports a cautious interpretation rather than a definitive causal claim.

### 3.3. Exploratory Fimasartan-Based Combination Analyses

Among fimasartan-based combinations, the crude 3-year MACE rates were nearly identical between fimasartan + CCB and fimasartan + thiazide users in the overall cohort (1200 of 34,711 [3.46%] vs. 273 of 7868 [3.47%], *p* = 0.9558). In treatment-naive patients, the corresponding rates were 3.13% and 3.38% (*p* = 0.676), and in treatment-maintenance patients, they were 3.60% and 3.79% (*p* = 0.5517). These comparisons should be considered exploratory because combination treatment choice may reflect clinical indications, blood pressure severity, and physician preference.

## 4. Discussion

In this nationwide cohort of Korean adults with hypertension and no established cardiovascular disease, the primary treatment-naïve new-user analysis showed a numerically lower risk of MACE with fimasartan than with CCBs, although the 95% confidence interval included the null value. Secondary analyses of the broader overall cohort and propensity-score-matched population showed stronger associations favoring fimasartan; however, these findings should be interpreted cautiously because residual confounding and selection bias cannot be excluded. The major contribution of the present study is not simply another ARB-versus-CCB comparison; rather, it evaluates fimasartan specifically in a large contemporary claims cohort and distinguishes this drug-specific evidence from prior ARB-class findings. The novelty of this study should be interpreted carefully. Prior nationwide data showed more favorable outcomes for ARBs than CCBs in a Korean simple-hypertension population [8]. The present study extends that work by focusing on fimasartan, an ARB with pharmacologic and clinical data demonstrating blood pressure-lowering efficacy [13,14,15,16,17,18,19,20,21]. It also complements recent real-world evidence showing that fimasartan combined with CCBs was associated with a lower need for treatment intensification than other ARB+CCB regimens [22]. Together, these data support the clinical relevance of studying individual ARBs rather than assuming that all ARBs have identical real-world outcome profiles. Several biologic hypotheses may explain why fimasartan could be associated with favorable outcomes, including high angiotensin II type 1 receptor affinity, sustained receptor binding, and potential anti-inflammatory or anti-atherosclerotic effects [14,26,27,28,29]. However, the present study cannot determine whether the observed associations were mediated by blood pressure reduction, differences in patient selection, treatment persistence, residual health behaviors, or unmeasured clinical factors. Although achieved blood pressure remained lower in the fimasartan group at 3-year follow-up, the absolute reductions in systolic and diastolic blood pressure were similar between the two treatment groups. The between-group difference in blood pressure was therefore already present at baseline and persisted during follow-up. Accordingly, residual confounding related to baseline blood pressure cannot be excluded. At the same time, post-treatment blood pressure may lie on the causal pathway between antihypertensive therapy and cardiovascular outcomes; therefore, adjustment for achieved blood pressure in the primary model could constitute overadjustment of a potential mediator. The large crude separation in MACE rates should therefore not be interpreted as the direct causal effect of selecting fimasartan rather than a CCB. Accordingly, the findings are best framed as real-world comparative effectiveness evidence that generates a clinically relevant hypothesis for prospective validation. The present analysis focused primarily on monotherapy to provide a more clearly defined comparison between fimasartan and CCB exposure and to reduce heterogeneity arising from multidrug regimens. However, contemporary hypertension management increasingly incorporates early combination therapy, particularly in patients requiring greater blood pressure reduction or with higher baseline cardiovascular risk. Recent real-world studies from Korea and other Asian populations have also documented the use of combination antihypertensive regimens in routine clinical practice. Accordingly, the present findings should primarily be interpreted in the context of patients managed with monotherapy and may not be directly generalizable to patients receiving initial or early combination treatment. Future studies should evaluate cardiovascular outcomes associated with fimasartan-based and CCB-based combination regimens [30,31]. The combination-therapy findings also merit cautious interpretation. Overall crude MACE rates were lower in fimasartan-based combination groups than in fimasartan monotherapy, but the comparison between fimasartan + CCB and fimasartan + thiazide showed no meaningful difference in the overall cohort or within treatment-naive and treatment-maintenance strata. This supports a pragmatic interpretation: when fimasartan is selected, the choice of add-on CCB or thiazide should remain individualized according to blood pressure control, comorbidities, tolerability, kidney function, metabolic profile, and adherence considerations. From a clinical perspective, the findings should not be interpreted as a recommendation to replace a well-tolerated and effective CCB regimen in an individual patient. Rather, they suggest that fimasartan has a favorable observed outcome profile when selected in routine Korean hypertension practice, and they support the value of evaluating individual ARBs instead of assuming uniform class effects. Treatment selection should continue to follow guideline-based assessment of blood pressure control, comorbid disease, kidney function, adverse-effect profile, drug interactions, cost, availability, and patient preference. The study has several strengths, including nationwide coverage, a very large sample size, a 3-year follow-up, prespecified exclusion of established cardiovascular disease, and propensity-score matching to improve comparability between groups. These features are particularly useful for evaluating relatively uncommon outcomes such as cardiac death and nonfatal MI in a broad hypertension population. This study also has important limitations. First, the observational design precludes causal inference, and residual confounding by indication is likely despite matching and multivariable adjustment. Fimasartan and CCB users differed substantially before matching, particularly in age and comorbidity profile. Second, claims data do not contain complete information on hypertension duration, office or home blood pressure trajectories, dose titration, medication adherence, drug discontinuation, lifestyle modification, salt intake, physical activity, or concurrent non-antihypertensive medications. Although prescription claims were used to define exposure, validated adherence metrics such as proportion of days covered or medication possession ratio were not available in the supplied dataset; differential adherence, persistence, treatment switching, or discontinuation could therefore have influenced the observed MACE differences. The treatment-maintenance subgroup may also be affected by survivor or immortal-time selection because persistence was defined using prescription coverage during follow-up; these subgroup findings should therefore be viewed as descriptive. Third, outcomes were identified using claims-based definitions rather than independent clinical adjudication, although combined diagnosis, hospitalization, procedure, and medication criteria were used where feasible to improve specificity; this is especially relevant for cardiac death and heart-failure hospitalization. Fourth, beta-blockers were not evaluated as a main comparator, and the exclusion of patients with prior cardiovascular disease means that the findings should not be generalized to patients with ischemic heart disease, heart failure, arrhythmias, or other compelling beta-blocker indications. Fifth, fimasartan is used predominantly in Korea, and the results may not generalize to populations with different ethnicities, health systems, prescribing patterns, or ARB availability. Finally, these findings should not be generalized to all ARBs; individual ARBs may differ in pharmacokinetic, pharmacodynamic, and real-world prescribing profiles [26].

## 5. Conclusions

In a nationwide Korean cohort of patients with hypertension and no established cardiovascular disease, fimasartan use was associated with a lower 3-year incidence of MACEs than CCB use. The association persisted after propensity-score matching and was supported by multivariable analyses, but the crude event-rate separation was interpreted descriptively because it was attenuated after adjustment. These results provide fimasartan-specific real-world evidence that is distinct from prior ARB-class comparisons, but prospective studies with detailed blood pressure, adherence, and adjudicated outcome data are needed before treatment-selection recommendations can be made.

## Figures and Tables

**Table 1:** Baseline demographic characteristics.

Characteristic	CCB (*n* = 934,534)	Fimasartan (*n* = 91,855)	*p* Value
Age, years	66.23 ± 11.55	60.82 ± 10.56	<0.0001
Age < 40 years	11,720 (1.25)	1722 (1.87)	<0.0001
Age 40–49 years	62,959 (6.74)	11,127 (12.11)	
Age 50–59 years	182,099 (19.49)	29,094 (31.67)	
Age 60–69 years	310,443 (33.22)	31,366 (34.15)	
Age 70–79 years	240,399 (25.72)	14,404 (15.68)	
Age ≥ 80 years	126,914 (13.58)	4142 (4.51)	
Male sex	490,028 (52.44)	52,250 (56.88)	<0.0001
Income < 25%	201,331 (21.54)	18,163 (19.77)	<0.0001
Income 25–75%	391,788 (41.92)	39,018 (42.48)	
Income > 75%	326,169 (34.90)	33,384 (36.34)	
Metropolitan residence	401,108 (42.92)	42,290 (46.04)	<0.0001
Systolic blood pressure, mmHg	133.17 ± 15.63	130.73 ± 15.54	<0.0001
Diastolic blood pressure, mmHg	82.50 ± 11.01	81.11 ± 10.98	<0.0001
Diabetes mellitus	131,280 (14.05)	15,791 (17.19)	<0.0001
Chronic kidney disease	7404 (0.79)	448 (0.49)	<0.0001
Charlson comorbidity index 0	703,054 (75.23)	69,386 (75.54)	<0.0001
Charlson comorbidity index 1	100,440 (10.75)	9274 (10.10)	
Charlson comorbidity index 2	96,390 (10.31)	10,236 (11.14)	
Charlson comorbidity index ≥ 3	34,650 (3.71)	2959 (3.22)	
Dyslipidemia	325,286 (34.81)	36,193 (39.40)	<0.0001
Current smoking	151,863 (16.25)	17,004 (18.51)	<0.0001
Height, cm	161.61 ± 9.42	163.86 ± 9.21	<0.0001
Weight, kg	66.17 ± 12.70	68.93 ± 13.01	<0.0001
BMI, kg/m^2^	25.20 ± 3.45	25.53 ± 3.44	<0.0001

Values mean ± standard deviation or number (%). BMI, body mass index; CCB, calcium channel blocker.

**Table 2:** Clinical event incidence during 3-year follow-up.

Outcome	CCB (*n* = 934,534)	Fimasartan (*n* = 91,855)	*p* Value
Primary outcome (MACE)	74,217 (7.94)	3877 (4.22)	<0.0001
All-cause death	14,140 (1.51)	340 (0.37)	<0.0001
Cardiac death	3072 (0.33)	44 (0.05)	<0.0001
Nonfatal myocardial infarction	10,375 (1.11)	517 (0.56)	<0.0001
Nonfatal stroke	50,922 (5.45)	3070 (3.34)	<0.0001
Secondary composite outcome	63,021 (6.74)	2893 (3.15)	<0.0001
Myocardial infarction	11,205 (1.20)	536 (0.58)	<0.0001
Revascularization	13,152 (1.41)	504 (0.55)	<0.0001
Hospitalization for heart failure	15,949 (1.71)	550 (0.60)	<0.0001
Ischemic stroke	33,854 (3.62)	1716 (1.87)	<0.0001

Values are number (%). MACE, major adverse cardiovascular event.

**Table 3:** Clinical events after propensity-score matching.

Outcome	CCB (*n* = 433,479)	Fimasartan (*n* = 86,694)	*p* Value
Primary outcome (MACE)	27,874 (6.43)	3774 (4.35)	<0.0001
All-cause death	4042 (0.93)	336 (0.39)	<0.0001
Cardiac death	834 (0.19)	44 (0.05)	<0.0001
Nonfatal myocardial infarction	4688 (1.08)	499 (0.58)	<0.0001
Nonfatal stroke	19,630 (4.53)	2988 (3.45)	<0.0001
Secondary composite outcome	24,139 (5.57)	2808 (3.24)	<0.0001
Myocardial infarction	4915 (1.13)	518 (0.60)	<0.0001
Revascularization	5810 (1.34)	482 (0.56)	<0.0001
Hospitalization for heart failure	5994 (1.38)	533 (0.61)	<0.0001
Ischemic stroke	11,889 (2.74)	1678 (1.94)	<0.0001

Values are number (%). MACE, major adverse cardiovascular event.

**Table 4:** Multivariable Cox models for major adverse cardiovascular events.

Population	Comparison	Adjusted HR (95% CI)	*p* Value
Overall cohort	fimasartan vs. CCB	0.851 (0.815–0.890)	<0.0001
Treatment-naive	fimasartan vs. CCB	0.895 (0.796–1.006)	0.0628
Treatment-maintenance	fimasartan vs. CCB	0.840 (0.800–0.881)	<0.0001

CCB was used as the reference category. CI, confidence interval; CCB, calcium channel blocker; HR, hazard ratio.

## Data Availability

Because we have analyzed the Korean National Health Insurance Service database, data sharing policy dependents on the Korean National Health Insurance Service. The health information data are only provided by Statistic analysis tool in “Data analysis room” located within the National Health Insurance Corporation in which PC for review and analysis of data is installed.

## Supplementary Materials

The following supporting information can be downloaded at: https://www.mdpi.com/article/10.3390/jcm15197669/s1, Table S1: Distribution of index antihypertensive prescriptions, with counts and percentages; Table S2: Baseline demographics after propensity-score matching; Table S3: Mean blood pressure values at baseline and 3-year follow-up; Table S4: Clinical event incidence in treatment-naive and treatment-maintenance groups; Table S5: Clinical event incidence after propensity-score matching; Table S6: Operational definitions, diagnosis codes, and procedure codes; Table S7: Distribution of individual calcium channel blocker agents in the propensity-score-matched cohort. Figure S1: Mean systolic and diastolic blood pressure changes from baseline to 3-year follow-up; Figure S2: Cumulative incidence rates of major adverse cardiovascular events during a 3-year follow-up; Figure S3: Cumulative incidence rates of major adverse cardiovascular events during a 3-year follow-up after propensity-score matching; Figure S4: Incidence of clinical events during 3-year follow-up after propensity matching.

## Abbreviations

The following abbreviations are used in this manuscript:

ARB	angiotensin II receptor blocker
BMI	body mass index
BP	blood pressure
CCB	calcium channel blocker
CCI	Charlson comorbidity index
CI	confidence interval
CKD	chronic kidney disease
HF	heart failure
HR	hazard ratio
ICD-10	International Classification of Diseases, Tenth Revision
MACE	major adverse cardiovascular event
MI	myocardial infarction
NHIS	National Health Insurance Service
