# Supplementary material for: Comparative Cardiovascular Outcomes of Fimasartan and Calcium Channel Blockers in Hypertension: A Nationwide 3-Year Follow-Up Cohort Study
Source: J Clin Med. 2026 Oct 3;15(19):7669. doi: 10.3390/jcm15197669 (PMC13666065; doi:10.3390/jcm15197669)

Supplementary materials

Supplementary Table S1. Distribution of index antihypertensive prescriptions, with counts and percentages.

Drug class or regimen	Treatment-naive (n=121,671)	Treatment-maintenance (n=1,442,269)
ARB(+ACEi)+CCB	28880 (23.74)	497854 (34.52)
ARB(+ACEi)+Thiazide	9067 (7.45)	202521 (14.04)
Fimasartan+CCB	1923 (1.58)	12061 (0.84)
ACEi	1541 (1.27)	9051 (0.63)
ARB	43380 (35.65)	341069 (23.65)
CCB	28326 (23.28)	323312 (22.42)
Fimasartan	6681 (5.49)	42348 (2.94)
Fimasartan+Thiazide	432 (0.36)	4342 (0.30)
Thiazide	1441 (1.18)	9711 (0.67)
Total	121671 (100.00)	1442269 (100.00)

Values are number (%). Percentages were calculated within each exposure-definition stratum.

Supplementary Table S2. Baseline demographics after propensity-score matching.

Variable	Category	CCB	CCB	Fimasartan	Fimasartan	P-value
		(N=433,479)	%	(N=86,694)	%	P-value
Age (years)	<40	7,803	1.80	1,532	1.77	0.916
	40-49	45,898	10.59	9,254	10.67	0.916
	50-59	130,745	30.16	26,198	30.22	0.916
	60-69	156,430	36.09	31,166	35.95	0.916
	70-79	71,986	16.61	14,402	16.61	0.916
	80~	20,617	4.76	4,142	4.78	0.916

Sex	Male	241,605	55.74	48,346	55.77	0.921
	Female	191,874	44.26	38,348	44.23	0.921
Income	< 25%	87,958	20.29	17,643	20.35	0.928
	25-75%	186,827	43.10	37,248	42.96	0.928
	> 75%	152,221	35.12	30,548	35.24	0.928
	NA	6,473	1.49	1,255	1.45	0.928
Region	Metropolitan	195,655	45.14	39,149	45.16	0.907
	Rural	237,824	54.86	47,545	54.84	0.907
Diabetes mellitus	No	366,026	84.44	72,936	84.13	0.952
	Yes	67,453	15.56	13,758	15.87	0.952
Chronic kidney disease	No	431,261	99.49	86,248	99.49	0.917
	Yes	2,218	0.51	446	0.51	0.917
Charlson comorbidity index	0	328,234	75.72	65,682	75.76	0.965
	1	43,907	10.13	8,764	10.11	0.965
	2	47,095	10.86	9,427	10.87	0.965
	3-	14,243	3.29	2,821	3.25	0.965
Dyslipidemia	No	270,155	62.32	53,821	62.08	0.982
	Yes	163,324	37.68	32,873	37.92	0.982
Smoking	No	353,568	81.57	70,786	81.65	0.955
	Yes	79,911	18.43	15,908	18.35	0.955
Height	Mean±SD	163.45±9.09	163.45±9.09	163.47±9.10	163.47±9.10	0.904
Weight	Mean±SD	68.36±12.68	68.36±12.68	68.39±12.72	68.39±12.72	0.957
BMI	18.5-22.9(Ref)	3,547	0.82	726	0.84	0.992
	<18.5	94,555	21.81	18870	21.77	0.992
	23-24.9	106,314	24.53	21184	24.44	0.992
	25-29.9	189,239	43.66	37884	43.7	0.992
	>29.9	39,824	9.19	8,030	9.26	0.992

Supplementary Table S3. Mean blood pressure values at baseline and 3-year follow-up.

Time point	Measure	CCB	Fimasartan	P-value
Baseline	SBP, mmHg	133.17 ± 15.63	130.73 ± 15.54	<.0001
	DBP, mmHg	82.50 ± 11.01	81.11 ± 10.98	<.0001
3-year follow-up	SBP, mmHg	130.83 ± 14.12	128.45 ± 14.02	<.0001
	DBP, mmHg	80.35 ± 9.82	79.00 ±9.84	<.0001
	Target SBP achievement rate, %	80.5	77.1	

Values are mean +/- standard deviation unless otherwise indicated.

Supplementary Table S4. Clinical event incidence in treatment-naive and treatment-maintenance groups.

Treatment-naive group

	CCB	Fimasartan	Fimasartan+CCB	Fimasartan+Thiazide	Thiazide	non-Fimasartan ARB	non-Fimasartan ARB+CCB	non-Fimasartan ARB+Thiazide	P-value
	(N=58,233)	(N=12,853)	(N=6,161)	(N=1,036)	(N=3,500)	(N=81,796)	(N=78,255)	(N=109)	P-value
**Primary outcome (MACEs)**	3322(5.70)	528(4.11)	193(3.13)	35(3.38)	195(5.57)	3566(4.36)	3028(3.87)	1(0.92)	<.0001
All-cause death	574(0.99)	54(0.42)	18(0.29)	4(0.39)	43(1.23)	433(0.53)	352(0.45)	0(0.00)	<.0001
Cardiac death	75(0.13)	6(0.05)	2(0.03)	0(0.00)	7(0.20)	75(0.09)	55(0.07)	0(0.00)	0.001
Non-fatal myocardial infarction	452(0.78)	83(0.65)	32(0.52)	11(1.06)	26(0.74)	579(0.71)	436(0.56)	0(0.00)	0.001
Non-fatal stroke	2347(4.03)	399(3.10)	146(2.37)	21(2.03)	126(3.60)	2599(3.18)	2290(2.93)	1(0.92)	0.001
**Secondary outcome**	3002(5.16)	427(3.32)	147(2.39)	24(2.32)	157(4.49)	3163(3.87)	2341(2.99)	1(0.92)	0.001
Myocardial infarction	481(0.83)	86(0.67)	32(0.52)	11(1.06)	31(0.89)	606(0.74)	458(0.59)	0(0.00)	0.001
Revascularization	650(1.12)	77(0.60)	22(0.36)	4(0.39)	17(0.49)	658(0.80)	384(0.49)	0(0.00)	0.001
Admission for heart failure	725(1.24)	92(0.72)	29(0.47)	5(0.48)	50(1.43)	881(1.08)	463(0.59)	0(0.00)	0.001
Ischemic stroke	1539(2.64)	224(1.74)	83(1.35)	8(0.77)	79(2.26)	1529(1.87)	1349(1.72)	1(0.92)	0.001

Treatment-maintenance group

	CCB	Fimasartan	Fimasartan+CCB	Fimasartan+Thiazide	Thiazide	non-Fimasartan ARB	non-Fimasartan ARB+CCB	non-Fimasartan ARB+Thiazide	P-value
	(N=376,344)	(N=44,380)	(N=12,771)	(N=4,586)	(N=18,294)	(N=360,551)	(N=523,742)	(N=2)	P-value
**Primary outcome (MACEs)**	23237(6.17)	1685(3.80)	460(3.60)	174(3.79)	1372(7.50)	16735(4.64)	21664(4.14)	0(0.00)	<.0001
All-cause death	3887(1.03)	187(0.42)	60(0.47)	21(0.46)	271(1.48)	2024(0.56)	3000(0.57)	0(0.00)	<.0001
Cardiac death	858(0.23)	36(0.08)	17(0.13)	1(0.02)	72(0.39)	353(0.10)	565(0.11)	0(0.00)	<.0001
Non-fatal myocardial infarction	2660(0.71)	217(0.49)	58(0.45)	19(0.41)	153(0.84)	2144(0.59)	2849(0.54)	0(0.00)	<.0001
Non-fatal stroke	17017(4.52)	1298(2.92)	347(2.72)	136(2.97)	957(5.23)	12768(3.54)	16081(3.07)	0(0.00)	<.0001

**Secondary outcome**	18305(4.86)	1200(2.70)	339(2.65)	133(2.90)	1121(6.13)	12601(3.49)	16361(3.12)	0(0.00)	<.0001
Myocardial infarction	2894(0.77)	225(0.51)	65(0.51)	19(0.41)	166(0.91)	2262(0.63)	3012(0.58)	0(0.00)	<.0001
Revascularization	2649(0.70)	186(0.42)	48(0.38)	19(0.41)	133(0.73)	2045(0.57)	2648(0.51)	0(0.00)	<.0001
Admission for heart failure	4085(1.09)	241(0.54)	80(0.63)	24(0.52)	296(1.62)	2726(0.76)	3495(0.67)	0(0.00)	<.0001
Ischemic stroke	11261(2.99)	682(1.54)	189(1.48)	83(1.81)	655(3.58)	7270(2.02)	9403(1.80)	0(0.00)	<.0001

Supplementary Table S5. Clinical event incidence after propensity-score matching.

Treatment-naive group

	**CCB**	**Fimasartan**	**P-value**
	(N=25,290)	(N=5,058)	
**Primary outcome (MACEs)**	1460(5.77)	245(4.84)	0.009
All-cause death	239(0.95)	28(0.55)	0.007
Cardiac death	32(0.13)	5(0.10)	0.607
Non-fatal myocardial infarction	187(0.74)	32(0.63)	0.413
Non-fatal stroke	1056(4.18)	189(3.74)	0.151
**Secondary outcome**	1324(5.24)	200(3.95)	0.000
Myocardial infarction	198(0.78)	34(0.67)	0.409
Revascularization	296(1.17)	32(0.63)	0.001
Admission for heart failure	311(1.23)	46(0.91)	0.054
Ischemic stroke	693(2.74)	111(2.19)	0.027

Treatment-maintenance group

	**CCB**	**Fimasartan**	**P-value**
	(N=147,791)	(N=29,554)	
**Primary outcome (MACEs)**	7585(5.13)	1343(4.54)	<.0001

All-cause death	963(0.65)	171(0.58)	0.151
Cardiac death	192(0.13)	34(0.12)	0.513
Non-fatal myocardial infarction	967(0.65)	148(0.50)	0.002
Non-fatal stroke	5770(3.90)	1037(3.51)	0.001
**Secondary outcome**	5925(4.01)	974(3.30)	<.0001
Myocardial infarction	1014(0.69)	155(0.52)	0.002
Revascularization	969(0.66)	146(0.49)	0.001
Admission for heart failure	1271(0.86)	195(0.66)	0.001
Ischemic stroke	3506(2.37)	583(1.97)	<.0001

Supplementary Table S6. Operational definitions, diagnosis codes, and procedure codes.

Domain	Definition	Codes or criteria
Screening	Hypertension	ICD-10 I10 in 2018
Exclusion	Heart failure	I50, I110, I130, I132
	Unstable angina	I20.0
	Angina pectoris	I20.1, I20.8, I20.9
	Atrial fibrillation	I48
	Stroke	I60-I66
	Peripheral artery disease	I70-I79, I70.2-I70.3, I70.9, I73.1, I73.8-I73.9; procedures M6597, M6605, M6613, M6632, M6620, O0161-O0171, O1643-O1644, O1645-O1646
	Prior myocardial infarction	I21-I23
Outcome	All-cause death	NHIS death records
	Cardiac death	Cardiovascular causes, I00-I99, including acute MI, stroke, heart failure, and sudden cardiac arrest
	Nonfatal myocardial infarction	Hospitalization claim with I21-I23, excluding fatal events
	Nonfatal stroke	Hospitalization claim with I60-I66, excluding fatal events
	Ischemic stroke	I63-I64

	Hospitalization for heart failure	Heart-failure diagnosis with concomitant diuretic treatment, ATC C03; dyspnea was not used as a separate claims criterion
	Percutaneous coronary intervention	M6551, M6552, M6561-M6567
	Coronary artery bypass graft surgery	O1640, O1641, O1647-O1649, OA640, OA641, OA647-OA649

Supplementary Table S7. Distribution of individual calcium channel blocker agents in the propensity-score-matched cohort.

Drug		(N=433,479 (%))
CCB	Amlodipine	336,581 (77.65)
	Nicardipine	22,655 (5.23)
	Nifedipine	21,113 (4.87)
	Diltiazem	11,375 (2.62)
	Lercanidipine	10,705 (2.47)
	Felodipine	7,580 (1.75)
	Verapamil	5,657 (1.31)
	Cilnidipine	4,575 (1.06)
	Benidipine	3,666 (0.84)
	Manidipine	3,261 (0.75)
	Lacidipine	2,776 (0.64)
	Nimodipine	1,848 (0.42)
	Barnidipine	953 (0.22)
	Dihydropyridine derivatives	502 (0.12)
	Nisoldipine	232 (0.05)

Supplementary Figure S1. Mean systolic and diastolic blood pressure changes from baseline to 3-year follow-up.

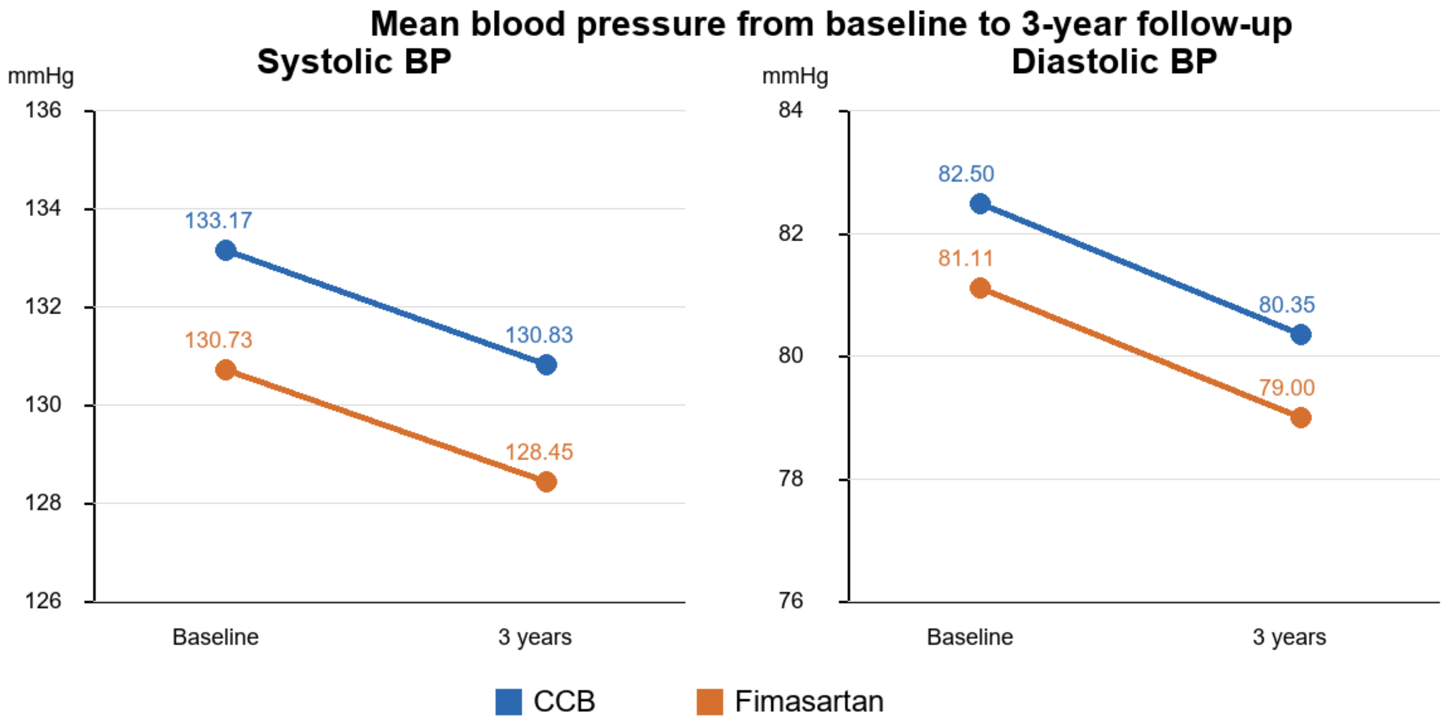

Supplementary Figure S2f-j. Cumulative incidence rates of major adverse cardiovascular events during 3-year follow-up. (f) Secondary outcome, (g) MI, (h) revascularization, (i) hospital admission for heart failure, (j) ischemic stroke.

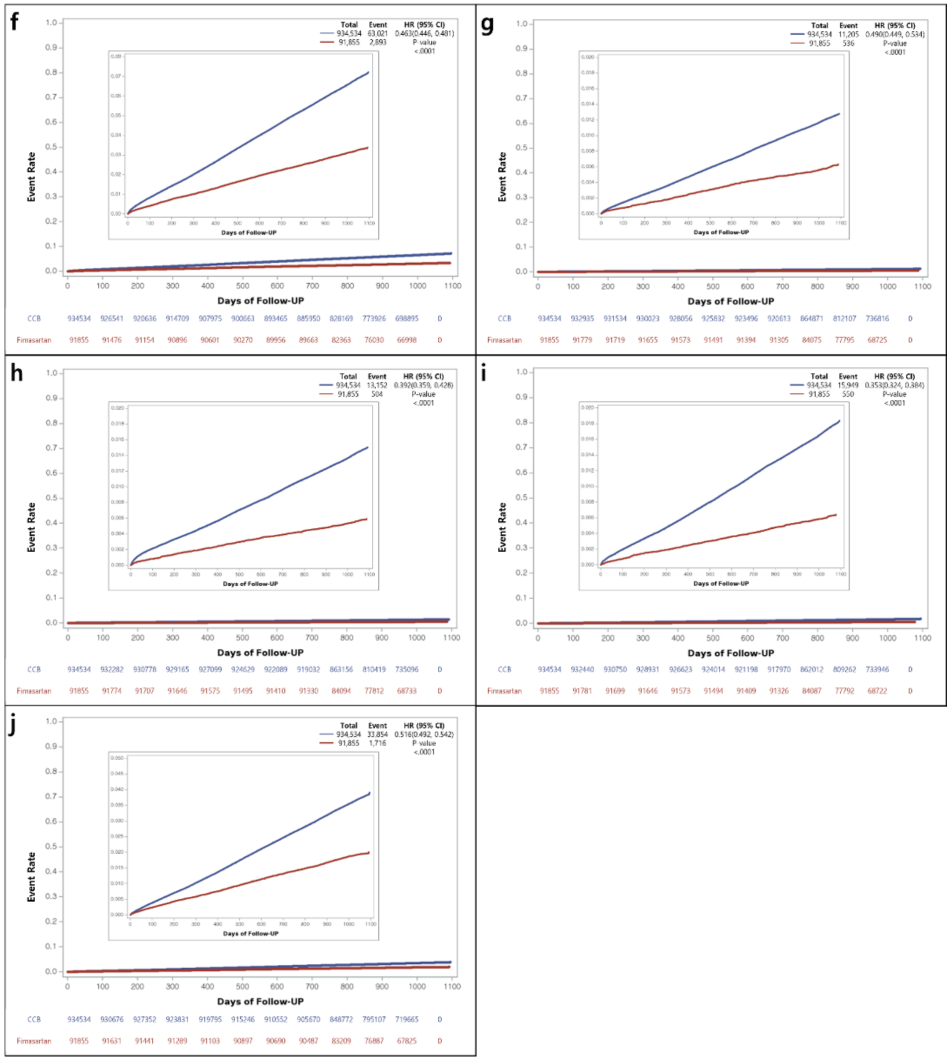

Supplementary Figure S3a-e. Cumulative incidence rates of major adverse cardiovascular events during 3-year follow-up after propensity score matching. (a) Major adverse cardiovascular events. (b) All-cause death. (c) Cardiac death. (d) Nonfatal MI. (e) Stroke.

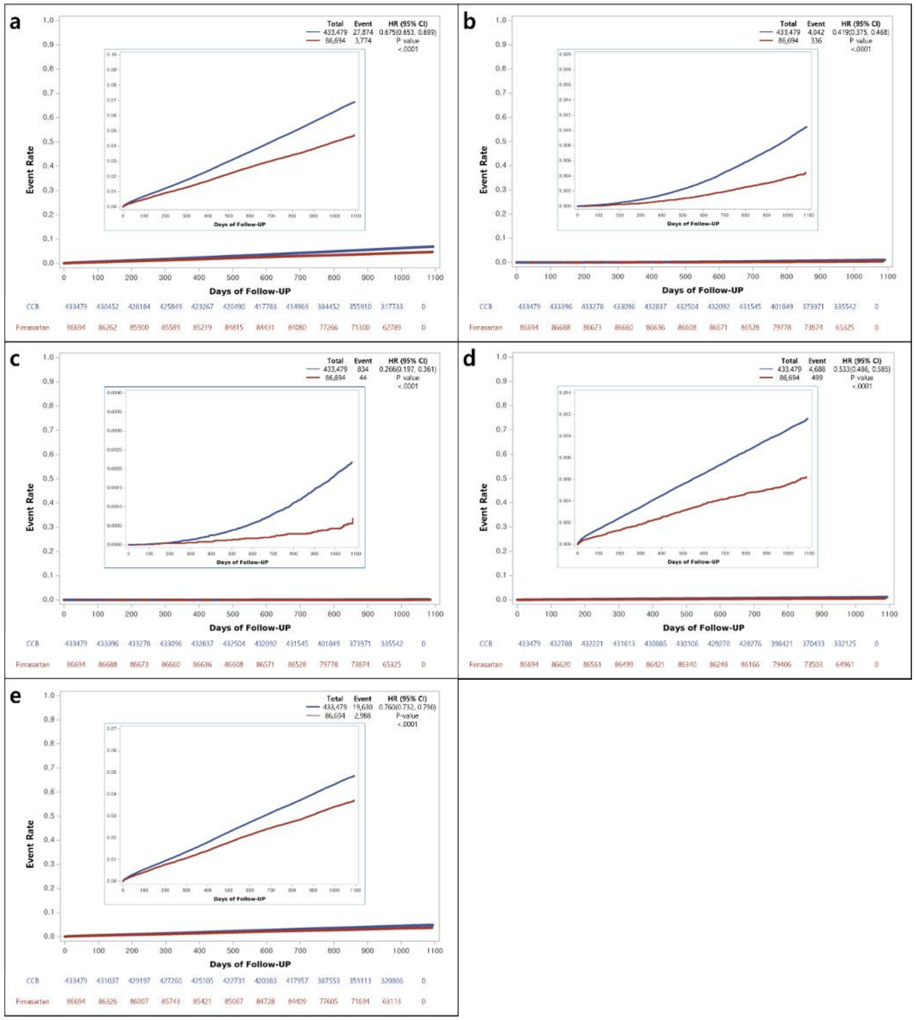

Supplementary Figure S4a-c. Incidence of clinical events during 3-year follow-up after propensity matching.

a

Subgroup	Fimasartan (N=91,855)	Fimasartan +CCB (N=34,711)	P-value	Odds Ratio, 95% CI
Primary outcome (MACEs)	3877(4.22)	1200(3.46)	<.0001	1.23 [1.15, 1.31]
All cause death	340(0.37)	109(0.31)	0.1340	1.18 [0.95, 1.46]
Cardiac death	44(0.05)	20(0.06)	0.4927	0.83 [0.49, 1.41]
Non-fatal myocardial infarction	517(0.56)	142(0.41)	0.0007	1.38 [1.14, 1.66]
Non-fatal Stroke	3070(3.34)	958(2.76)	<.0001	1.22 [1.13, 1.31]
Secondary outcome	2893(3.15)	837(2.41)	<.0001	1.32 [1.22, 1.42]
Myocardial infarction	536(0.58)	150(0.43)	0.0011	1.35 [1.13, 1.62]
Revascularization	504(0.55)	91(0.26)	<.0001	2.10 [1.68, 2.62]
Admission from heart failure	550(0.60)	139(0.40)	<.0001	1.50 [1.24, 1.81]
Ischemic stroke	1716(1.87)	539(1.55)	0.0002	1.21 [1.09, 1.33]

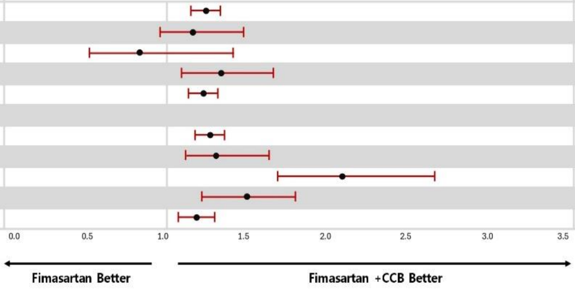

b

Subgroup	Fimasartan (N=91,855)	Fimasartan +Thiazide (N=7,868)	P-value	Odds Ratio, 95% CI
Primary outcome (MACEs)	3877(4.22)	273(3.47)	0.0014	1.23 [1.08, 1.39]
All cause death	340(0.37)	31(0.39)	0.7387	0.94 [0.65, 1.36]
Cardiac death	44(0.05)	4(0.05)	0.7896	0.94 [0.34, 2.62]
Non-fatal myocardial infarction	517(0.56)	33(0.42)	0.0992	1.34 [0.94, 1.91]
Non-fatal Stroke	3070(3.34)	211(2.68)	0.0016	1.25 [1.09, 1.45]
Secondary outcome	2893(3.15)	197(2.50)	0.0015	1.27 [1.09, 1.47]
Myocardial infarction	536(0.58)	34(0.43)	0.0873	1.35 [0.96, 1.91]
Revascularization	504(0.55)	23(0.29)	0.0026	1.88 [1.24, 2.86]
Admission from heart failure	550(0.60)	37(0.47)	0.1527	1.27 [0.91, 1.78]
Ischemic stroke	1716(1.87)	120(1.53)	0.0299	1.23 [1.02, 1.48]

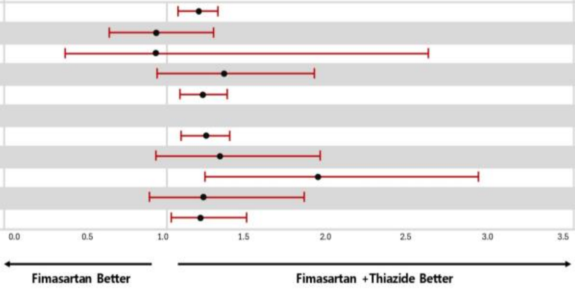

c

Subgroup	Fimasartan +CCB (N=34,711)	Fimasartan +Thiazide (N=7,868)	P-value	Odds Ratio, 95% CI
Primary outcome (MACEs)	1200(3.46)	273(3.47)	0.9558	1.00 [0.87, 1.14]
All cause death	109(0.31)	31(0.39)	0.2632	0.80 [0.53, 1.19]
Cardiac death	20(0.06)	4(0.05)	1.0000	1.13 [0.39, 3.32]
Non-fatal myocardial infarction	142(0.41)	33(0.42)	0.8971	0.98 [0.67, 1.43]
Non-fatal Stroke	958(2.76)	211(2.68)	0.7016	1.03 [0.89, 1.20]
Secondary outcome	837(2.41)	197(2.50)	0.6304	0.96 [0.82, 1.13]
Myocardial infarction	150(0.43)	34(0.43)	0.9999	1.00 [0.69, 1.45]
Revascularization	91(0.26)	23(0.29)	0.6402	0.90 [0.57, 1.42]
Admission from heart failure	139(0.40)	37(0.47)	0.3835	0.85 [0.59, 1.22]
Ischemic stroke	539(1.55)	120(1.53)	0.8576	1.02 [0.83, 1.24]

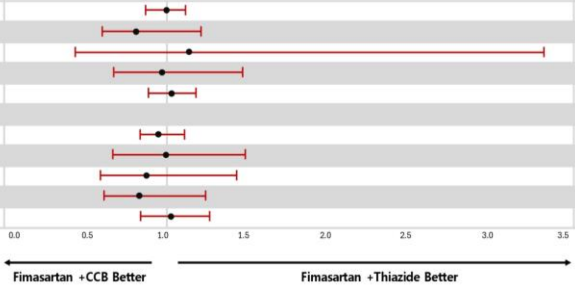

Supplement: Supplementary file 1 [file jcm-15-07669-s001.zip › jcm-4565623-supplementary.pdf]
